# Effects of dutasteride and tamsulosin on penile morphology in a rodent model

**DOI:** 10.1590/S1677-5538.IBJU.2022.0583

**Published:** 2023-04-15

**Authors:** Marcello H. A. Da Silva, Waldemar S. Costa, Francisco J. B. Sampaio, Diogo B. de Souza

**Affiliations:** 1 Unidade de Pesquisa Urogenital Universidade do Estado do Rio de Janeiro Rio de Janeiro RJ Brasil Unidade de Pesquisa Urogenital, Universidade do Estado do Rio de Janeiro - Uerj, Rio de Janeiro, RJ, Brasil

**Keywords:** Erectile Dysfunction, Prostatic Hyperplasia, Dutasteride

## Abstract

**Purpose:**

To evaluate the penile morphology after the isolated and combined administration of dutasteride and tamsulosin in a rodent model.

**Materials and Methods:**

Forty male rats were assigned into the following groups: Control group (C, receiving distilled water, n=10); Dutasteride group (D, receiving 0.5 mg/Kg/day of dutasteride, n=10); Tamsulosin group (T, receiving 0.4 mg/Kg/day of tamsulosin, n=10); and Dutasteride associated with Tamsulosin group (DT, receiving both drugs n = 10). All drugs were administered via oral gavage. After 40 days, the animals were submitted to euthanasia and their penises were collected for histomorphometric analyses. Data were compared using one-way ANOVA followed by *Bonferroni’s* post-test, considering p<0.05 as significant.

**Results:**

The sinusoidal space and smooth muscle fiber surface densities (Sv), and the cross-sectional penile areas of rats in groups D, T and DT were reduced in comparison to controls with the most notable reductions in the combined therapy group. The connective tissue and elastic system fibers Sv were augmented in groups D, T and DT in comparison with the control group, again with the most pronounced changes observed in animals receiving the combined therapy.

**Conclusion:**

Both treatments with dutasteride or tamsulosin promoted penile morphometric modifications in a rodent model. The combination therapy resulted in more notable modifications. The results of this study may help to explain the erectile dysfunction observed in some men using these drugs.

## INTRODUCTION

Benign prostatic hyperplasia (BPH) affects 50% of men older than 50 years old and 90% of men in their 80s (1-3). Enlargement of the prostatic epithelial and stromal tissues constricts the prostatic urethra, resulting in manifestations commonly known as lower urinary tract symptoms (LUTS) (1).

The first-line pharmacological treatment for BPH includes 5-alpha reductase inhibitors (5-ARIs) (4, 5). This class of drugs prevents the conversion of testosterone to dihydrotestosterone (DHT), which is the most active androgen (1). As an androgen-dependent organ, the prostate volume is commonly reduced by DHT depletion, which ameliorates the clinical symptoms associated with BPH (1, 2). However, some patients still present with LUTS while on 5-ARIs treatment (6), and adverse effects is also an important issue associated with this treatment option. Erectile dysfunction and decreased libido, with morphological alterations in the corpus cavernosum (CC) have been previously described (7-10). Of special importance, the odds-ratio to develop erectile disfunction when using dutasteride has been calculated as 1.47 (6).

Another pharmacological option for the BPH treatment is the use of tamsulosin which is an alpha-1-blocker. This drug relaxes the prostatic stromal smooth muscle, helping with LUTS in these patients (11). However, treatment with tamsulosin alone may not be sufficient to improve the clinical symptoms and is associated with hypotension, retrograde ejaculation, and other adverse effects (11).

The combined use of dutasteride (a 5-ARI) and tamsulosin has emerged as a therapeutic option to improve treatment efficacy and reduce the adverse effects (12,13). Although the combined treatment is associated with better preservation of erectile function (12, 14, 15), it is still unknown if penile histoarchitecture is conserved after the use of dutasteride and tamsulosin. Knowledge regarding the effects of these (routinely used) drugs on penile morphology is important as it adds information to urological literature and provide a scientific basis for clinical decisions.

The hypothesis of this study is that the use of combined therapy (with dutasteride and tamsulosin) may result in fewer morphological alterations than the isolated use of these drugs. Thus, the aim of this study is to evaluate, in a rodent model, the penile morphology after isolated and combined administration of dutasteride and tamsulosin.

## MATERIALS AND METHODS

This project was formally approved by the local ethics committee under the protocol number CEUA-057/2018 and was conducted in accordance with the national and international regulations on animal experimental use.

Forty male Wistar rats were used in this study. All animals were bred in the Urogenital Research Unit`s animal facilities and were included in the experiment after completing four months of age. They were kept in a room with a controlled temperature (22°C ± 1°C) and artificial dark-light cycles (lights on from 7:00 am to 7:00 pm) and had free access to standard rat food and water.

Animals were divided into the following groups: Control group (C, n = 10); Dutasteride group (D, n = 10); Tamsulosin group (T, n = 10); and Dutasteride associated with Tamsulosin group (DT, n = 10). Animals of group C received distilled water each morning. Group D received 0.5 mg/Kg/day of dutasteride (Avodart™, GlaxoSmithKline Pharmaceuticals S.A., Poznan, Polonia) (8, 9). Rats of group T received 0.4 mg/Kg/day of tamsulosin (Secotex™, Astellas Pharma, Meppel, Netherlands) (16, 17). Finally, group DT received 0.5mg/Kg/day of dutasteride and 0.4mg/Kg/day of tamsulosin (Combodart™, GlaxoSmithKline Pharmaceuticals S.A., Poznan, Polonia). All drugs were administered by gavage diluted in sterile water to 3 mL of final volume, during 40 consecutive days.

After 40 days, the animals were submitted to euthanasia by isoflurane (Forane™, Abbott Laboratories, Buenos Aires, Argentina) inhalation in an induction chamber. The animals were weighed at the beginning of the study and immediately before euthanasia. The penis of each animal was collected, and its skin-denuded middle shaft was fixed in 4% buﬀered formaldehyde solution. Samples were routinely processed for paraffin embedding and 5µm-thick sections were used for histomorphometric evaluations (8, 9, 18).

The cross-sectional penile area, the area of CC (including its tunica albuginea), and the area of CC without the tunica albuginea were evaluated in Masson’s trichrome stained sections. For this purpose, 5 images, separated by (at least) 100µm, were captured under 20× magnification by a digital camera (Axiocam 506 color, Carl Zeiss, Jena, Germany) coupled to a stereomicroscope (Discovery V.8, Carl Zeiss). These areas were measured using the “polygons” tool of the Image J software (version 1.45s, National Institutes of Health, Bethesda, USA), and expressed in mm2. The area of the tunica albuginea was calculated as the difference between the CC area with and without its tunica albuginea (8, 9).

The surface density (Sv) of the CC connective tissue, sinusoidal space, smooth muscle fibers, and elastic system fibers were measured using the point-counting method (19). Briefly, a 100-point grid was superimposed over the images using the Image J software, and each structure “touched” by a point was counted. The result, expressed as a percentage, was calculated after measuring 25 images from different randomly captured fields for each animal (18, 19).

**Figure 1:**
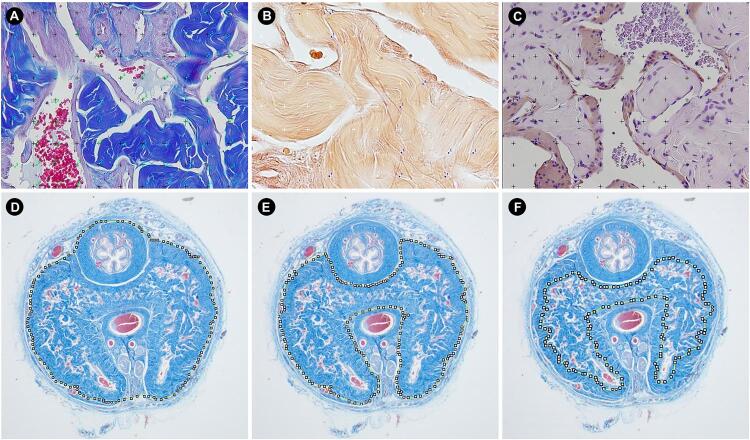
Illustrations of the morphometrical measures used in the study. A) presents Masson’s trichrome stained section whereas the surface density of connective tissue and sinusoidal space are being analyzed (in blue and green dots, respectively). B) presents Weigert's resorcin-fuchsin stained section whereas the surface density of elastic system fiber is being analyzed (in blue dots). C) presents anti-alpha-actin immunostained section whereas smooth muscle is being analyzed (in blue dots). D, E and F) presents penile cross-section stained by Masson’s trichrome whereas the cross-sectional penile area (D), area of corpus cavernosum with tunica albuginea (E), and area of corpus cavernosum without tunica albuginea (F) are being analyzed.

Each structure was assessed using an appropriate histochemical or immunohistochemical method and magnification. The Sv of connective tissue and sinusoidal spaces were assessed on Masson’s trichrome stained sections captured at 400x magnification (20). The Sv of smooth muscle fibers was measured on immunolabeled sections. For this purpose, an anti-alpha-actin IgG monoclonal primary antibody (Cat No A2547, Sigma-Aldrich, St. Louis, USA) was used. This antibody was diluted by 1:400 and incubated for 12 hours. Further, a secondary antibody and biotin-streptavidin kit (Histostain™, Invitrogen, Camarillo, USA) was applied following the manufacturer’s instructions. These smooth muscle immunolabeled sections were captured at 400× magnification (19). To assess the Sv of elastic system fibers, histological sections were stained with Weigert’s resorcin-fucsin method (with previous oxidation), and images were captured at 600x magnification (21). Figure-1 illustrates the morphometrical methods used.

Further, the CC collagen distribution and types were assessed in picrosirius red stained sections, observed at 400× magnification using polarization microscopy. Under polarized light and with this histochemical technique, it is possible to observe the collagen fibers birefringence and differentiate collagen types I (red/orange) and III (green) (20). All images used for collagen and for Sv analyses were captured using a digital camera (DP70, Olympus, Tokyo, Japan) coupled with a microscope (BX51, Olympus).

All morphometric data were considered as parametric values by Kolmogorov-Smirnov normality test. Considering the number of groups, the data were analyzed using one-way ANOVA. Bonferroni’s post-test was used to compare the means between the groups. Statistically significant differences were considered at p < 0.05. All results were presented as mean ± standard deviation. All statistical analyses were performed using GraphPad Prism version 5.0 (GraphPad Software, San Diego, USA).

## RESULTS

The weights of the animals at the beginning and at the end of the experiment were similar among all groups. Regarding the cross-sectional penile area, all groups that received drugs showed reduced values in comparison to that of control animals. Group D had a 16.1% reduction, and group T had a 17.6% reduction in the cross-sectional penile area compared to that of group C. The penises of animals from group DT showed a more pronounced reduction in cross-sectional area of 26.7% than those of group C did. However, the mean area in groups D, T, and DT were statistically similar (all morphometrical data is presented in Table-1 and the raw data is presented in supplementary Table -1).

**Table 1:** – Morphometrical data of rats receiving dutasteride, tamsulosin or the association of both.

	C	D	T	DT	p value*
Initial body weight (g)	286.6 ± 9.0	287.8 ± 21.5	288.0 ± 9.5	293.4 ± 13.4	0.724
Final body weight (g)	321.5 ± 5.4	333.4 ± 9.2	324.8 ± 11.0	322.1 ± 16.9	0.094
Cross-sectional penile area (mm^2^)	4.83 ± 0.56	4.05 ± 0.25 ^a^	3.98 ± 0.41 ^a^	3.54 ± 0.23 ^a^	<0.0001
Area of the corpus cavernosum - including tunica albuginea (mm^2^)	3.33 ± 0.34	2.79 ± 0.19 ^a^	2.84 ± 0.32 ^a^	2.39 ± 0.18 ^a, b, c^	<0.0001
Area of the corpus cavernosum - without tunica albuginea (mm^2^)	2.00 ± 0.17	1.90 ± 0.09	1.77 ± 0.24 ^a^	1.56 ± 0.14 ^a, b^	<0.0001
Area of the tunica albuginea (mm^2^)	1.33 ± 0.24	0.96 ± 0.04 ^a^	1,07 ± 0.13 ^a^	0.88 ± 0.10 ^a^	<0.0001
Connective tissue Sv (%)	46.44 ± 3.75	66.69 ± 4.23 ^a^	61.44 ± 5.5^7^ a	70.08 ± 3.64 ^a, c^	<0.0001
Sinusoidal space Sv (%)	30.18 ± 4.85	21.38 ± 3.44 ^a^	22.13 ± 3.77 ^a^	19.02 ± 2.96^a^	<0.0001
Smooth muscle fibers Sv (%)	22.12 ± 1.94	10.90 ± 1.35 ^a^	15.43 ± 2.48 ^a^	9.90 ± 1.37 ^a, c^	<0.0001
Elastic system fibers Sv (%)	12.44 ± 2.66	19.25 ± 2.08^a^	14.25 ± 1.82	19.80 ± 2.51 ^a, c^	<0.0001

C: Control group; D: Dutasteride group; T: Tamsulosin group; DT: Dutasteride associated with Tamsulosin group; Sv: Surface density.

* p value represents the ANOVA results. Bonferroni’s post test results are signalized by: a when different from C; b when different from D; and c when different from T.

Data expressed as mean ± standard deviation.

The area of CC with tunica albuginea was also reduced in groups D and T, by 16.2% and 14.7%, respectively, in comparison to that in group C. Again, group DT showed a more pronounced reduction in CC area with tunica albuginea of 28.2% than group C rats did. Group DT also had a smaller cavernosal area than groups D and T did.

**Figure 2:**
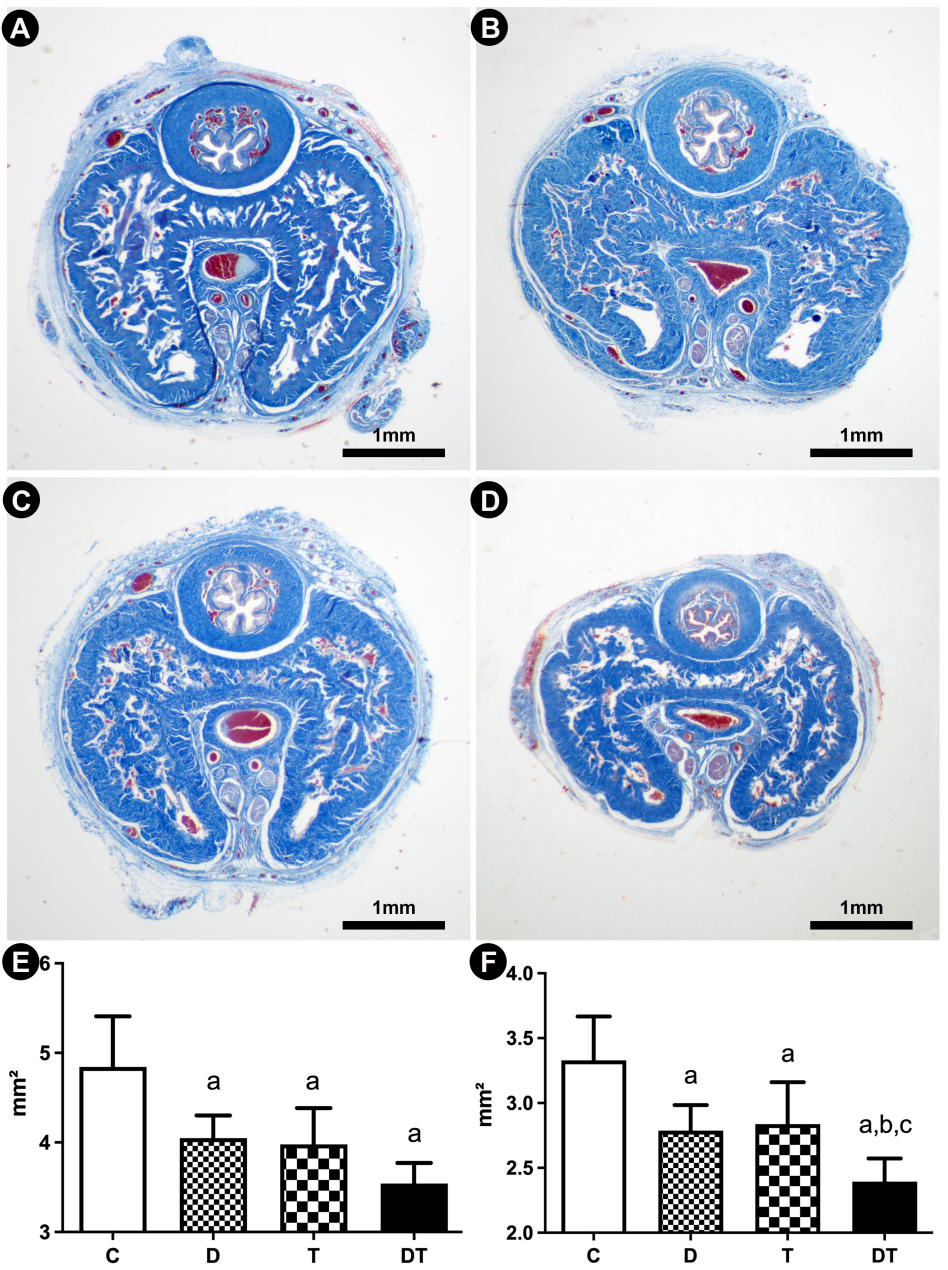
Photomicrographs of penile cross-sections illustrating the modifications in penile and cavernosal areas in response to administration of dutasteride (B), tamsulosin (C), and combined therapy (D), in comparison to control animals (A). Masson’s trichrome, 20x. Graphics illustrates the statistical differences on cross-sectional penile area (E) and Area of corpus cavernosum with tunica albuginea (F). Groups marked with “a” are different from group C. Groups marked with “b” are different from group D. Groups marked with “c” are different from group T.

The area of CC without the tunica albuginea did not differ between groups D and C. Groups T and DT had reductions of 11.5% and 22.0% in comparison to C for this parameter. The CC area without tunica albuginea in group DT was 11.8% smaller than that in group T. The calculated area of tunica albuginea was reduced by 27.8%, 19.5%, and 33.8% in groups D, T and DT (respectively), in comparison to group C. Regarding this parameter, no difference was found among these three treated groups. Figure-2 illustrates the findings regarding the evaluated areas of the penis and CC.

The connective tissue Sv of CC was 43.6% higher in group D, and 32.3% higher in group T in comparison to group C. Again, group DT shower a more drastic alteration, with 50.9% higher values than group C. Group DT was also considered statistically different from that in group T, with a 14.0% higher mean.

Regarding the Sv of sinusoidal space, groups D, T and DT had reductions of 29.1%, 26.7%, and 37.0% (respectively), then that in group C. For this parameter, no difference was observed among the three treated groups.

**Figure 3:**
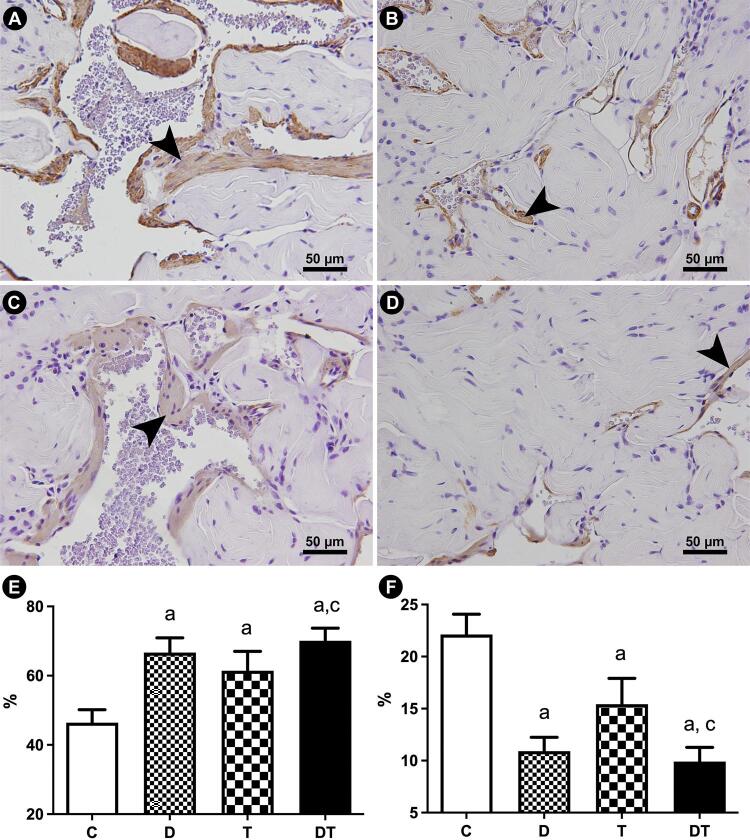
Photomicrographs of penile corpus cavernosum illustrating the modifications in smooth muscle fibers, and connective tissue content in response to administration of dutasteride (B), tamsulosin (C), and combined therapy (D), in comparison to control animals (A). Arrowheads indicates the smooth muscle fibers. Anti-alpha-actin immunostaining, 400x. Graphics illustrates the statistical differences on Connective tissue surface density (E) and Smooth muscle fibers surface density (F). Groups marked with “a” are different from group C. Groups marked with “b” are different from group D. Groups marked with “c” are different from group T.

The smooth muscle fibers Sv was reduced by 50.7% in group D in comparison to group C. Rats treated with tamsulosin showed a more discrete reduction (by 30.2%) in cavernosal musculature. On the other hand, group DT showed a more drastic reduction, by 55.2%, in comparison to group C. Group DT was also considered different from group T, with 35.8% lower smooth muscle content. Figure-3 illustrates the findings regarding the smooth muscle fibers Sv.

**Figure 4:**
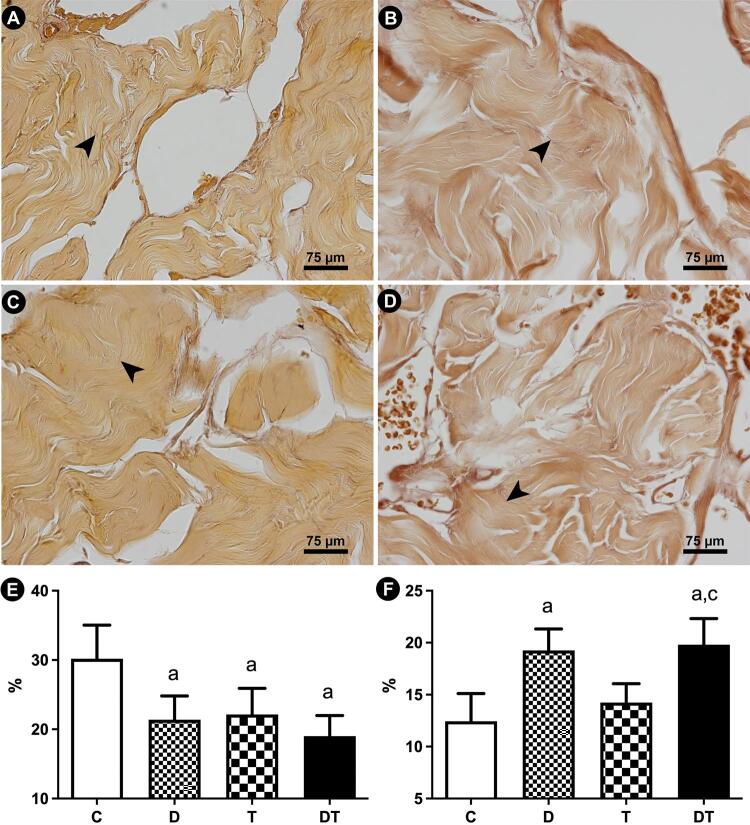
Photomicrographs of penile corpus cavernosum illustrating the modifications in elastic system fibers and sinusoidal space in response to administrations of dutasteride (B), tamsulosin (C), and combined therapy (D), in comparison to control animals (A). Arrowheads indicates the elastic system fibers. Weigert’s resorcin-fuchsin, 600x. Graphics illustrates the statistical differences on Sinusoidal space surface density (E) and elastic system fibers surface density (F). Groups marked with “a” are different from group C. Groups marked with “b” are different from group D. Groups marked with “c” are different from group T.

The elastic system fibers Sv were 54.7% higher in group D than in group C. Again, in this parameter group T was less affected with the treatment, with statistically similar values to that in group C. One more time, group DT was more affected with a 59.1% higher mean than group C. Differences among groups DT and T was also observed, with the first 38.9% higher than the animals treated with tamsulosin alone. The findings regarding elastic system fibers Sv are illustrated in Figure-4.

**Figure 5:**
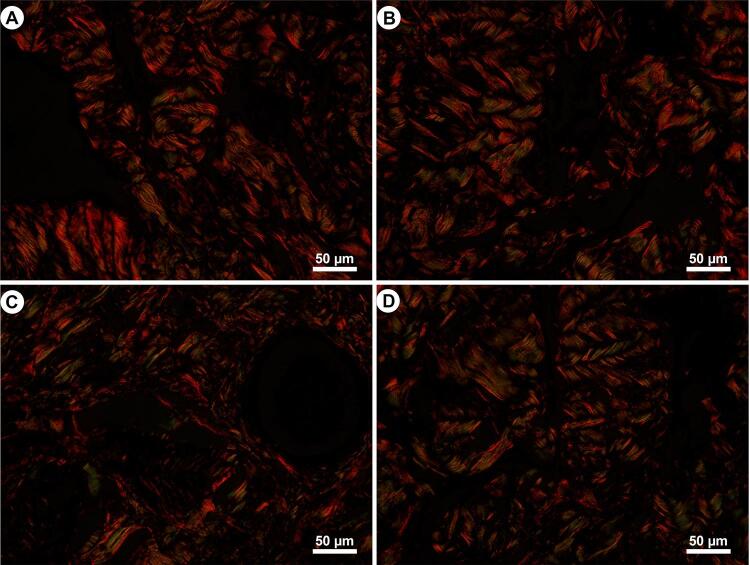
Photomicrographs of penile corpus cavernosum illustrating the collagen types as seen in polarized microscopy in animals receiving dutasteride (B), tamsulosin (C), and combined therapy (D), in comparison to control animals (A). Picrosirius red staining method, observed at 400× magnification by polarization microscopy.

Regarding the CC collagen analysis, it was observed similar distribution among all groups. Most fibers were observed in reddish color, characterizing the predominance of type I collagen in CC Figure-5 illustrates the findings regarding the collagen analysis.

## DISCUSSION

Despite the beneficial effects of dutasteride in BPH treatment, it is well known that this drug is associated with erectile dysfunction (6). Previous studies of our group have shown that dutasteride leads to important morphological modifications on the CC of rodents (8, 9). The mechanisms by how 5-ARIs alters penile function is admitted being hormonal, by depleting DHT. As an androgen-dependent organ, a decrease in male hormone levels is associated with functional and morphological prejudice. DHT is also involved in the local synthesis of nitric oxide, which plays an important role in cavernosal smooth muscle relaxation which is necessary for penile erection (7).

Tamsulosin has emerged as a treatment option for BPH and LUTS owing to its different mode of action. One notable advantage of this alpha-1-blocker is to not interfere with erectile function or testosterone levels of patients (22, 23). However, in some patients, combination therapy (with dutasteride and tamsulosin) is necessary to adequately treat BPH and LUTS. Although the effects of dutasteride on the cavernosal tissue of experimental animals have been previously reported, this is the first study to report penile morphological alterations caused by tamsulosin (alone) or in combination with dutasteride.

Reductions in penile cross-sectional and cavernosal areas were observed in all groups that received the drugs. Overall, for these measurements, the tamsulosin-treated animals showed slightly worse results than those that received dutasteride. As the effects of tamsulosin on penile size or diameter have never been studied, neither in patients nor in experimental models, these results were unexpected. Thus, it is somehow difficult to imagine possible mechanisms that explain these findings. One possible explanation is that the alpha‐adrenoceptor blockade leads to a (already known) reduced blood pressure (24, 25), which may lead to reduced penile blood flow. This altered penile blood flow may have led to cavernosal morphological modifications.

In the clinical scenario, tamsulosin has been associated with priapism (26). One possible explanation is that the alpha-receptor blockade would inhibit the sympathetic effects on cavernous tissue, which are necessary for detumescence (26). Although this was not the main objective of the present study, these results may be linked to tamsulosin-induced priapism. Continuous sympathetic blockade may have caused the morphological alterations observed in the animals of group T.

Other modifications in the cavernosal histoarchitecture were also observed in groups D, T and DT. The dutasteride-treated animals in this study confirmed that 5-ARIs induces penile fibrosis (higher connective tissue and lower smooth muscle content in CC) (8, 9). Interestingly, animals receiving tamsulosin also showed similar alterations. Even so, the results of connective tissue, sinusoidal space, and smooth muscle content of Group T were less drastically altered than those of group D. The exception to be mentioned was regarding the elastic system fibers content, which was not altered by tamsulosin administration, but was altered by dutasteride.

Most importantly, the combined use of the drugs proved to be (for all analyzed parameters) more deleterious to penile morphology than dutasteride or tamsulosin administration alone. Thus, it is possible to assume that the drugs had an addictive effect on the cavernosal tissue. Further, it may be presumed that the drugs act via different mechanisms on penile morphology.

As in most organs, and penises are not different on this aspect, morphology is closely related to function. Specifically in respect of the masculine genital organs, adequate amounts of each cavernosal tissue are critical for achieving and maintaining an erection. This has been observed in both humans (27) and experimental animals (20, 28, 29).

The CC (of both man and rodents) is basically composed of smooth muscle fibers, connective tissue, sinusoidal space, and blood vessels; each of these tissues has its own characteristics and functions. During an erection, the smooth muscle (in response to neural stimuli) relaxes, and the sinusoidal space becomes fulfilled with blood. The connective tissue (which is mainly composed by collagen and elastic fibers) must permit the penile enlargement and elongation while should also restrain its expansion (what maintains a high-pressure environment). Furthermore, all components must exhibit elasticity to restore normal penile morphology after the erection estate (20). For this complicated physiological mechanism to occur, adequate proportion of each cavernosal tissue is necessary for regular erection and detumescence (27).

It is with this concept in mind that it becomes very interesting to quantify each cavernosal tissue. The use of morphometric methods in erectile dysfunction research permits the accurate comparison of specimens subjected to different conditions. Furthermore, the surface density of connective tissue, smooth muscle fibers, and elastic system fibers are commonly assessed for this purpose (19). This method has been successfully used to determine the proportions of CC components in various situations (8, 18, 20, 28 ,30). To the best of our knowledge, this study is the first of its kind to show the cavernosal modifications after tamsulosin, either alone or in combined administration with dutasteride.

One aspect that requires further investigation is the persistence of these modifications; whether the change is permanent or can be restored after treatment discontinuation. Future studies focusing on the long-term effects of these drugs (either continued or discontinued) are warranted. A comparison of the effects of pharmacological treatments with those of non-pharmacological BPH options also requires further investigation. The effect of prostate resection on penile morphology is unknown. Recently, new minimally invasive treatment options have been developed; the effects of these techniques on penile morphology need to be studied.

This study provides information that may help to understand clinical urological problems. The use of 5-ARIs to treat BPH is sometimes associated with severe side-effects. The results of this study reinforce that erectile dysfunction after dutasteride administration are a consequence of morphological modification of penile structures. As dutasteride is now being used (as well as finasteride) for androgenic alopecia treatment, is expected that more patients will present to urologists with 5-ARI side-effects.

As limitations of the study, it should be pointed out that these results were obtained under experimental conditions that are different from the clinical scenario. However, these findings highlight the incidence of penile dysfunction associated with dutasteride and tamsulosin therapy. Although rodent penises show similar structural components and responses to human penises (18, 20, 31), they have different structural organizations. Furthermore, the study rats did not have erectile dysfunction or BPH and different results may be obtained in these conditions. The age of the animals used corresponded to that of adult individuals; however, it cannot accurately be transposed to human age. As this is an important factor in penile responses, it could be altered in the clinical setting. The age of the animals used corresponded well with that of patients using 5-ARIs for androgenic alopecia. In summary, the present study may be a landmark, providing a better understanding of the effects of alpha-1-blocker therapy.

## CONCLUSIONS

Dutasteride or tamsulosin treatment promoted penile morphological modifications in a rodent model. Dutasteride induced more prominent modifications than tamsulosin did. Combined therapy with both drugs did not prevent the effects of dutasteride. On the contrary, it resulted in more pronounced modifications. Future studies to elucidate the possible mechanisms by which tamsulosin affects penile morphology and function are necessary.

## APPENDIX

Supplementary table 1 - Raw data of animals after dutasteride, tamsulosin or the association of both drugs administration.


