# Supplementary material for: Effects of dutasteride and tamsulosin on penile morphology in a rodent model
Source: Int Braz J Urol. 2023 Apr 15;49(3):320–33. doi: 10.1590/S1677-5538.IBJU.2022.0583 (PMC10335897; doi:10.1590/S1677-5538.IBJU.2022.0583)
Supplement: Supplementary file 1 [file 1677-6119-ibju-49-03-0320-suppl01.pdf]

APPENDIX

Supplementary table 1 - Raw data of animals after dutasteride, tamsulosin or the association of both drugs administration.

Animals from Control Group	Initial body weight (g)	Final body weight (g)	Cross-sectional penile area (mm²)	Area of the corpus cavernosum - including tunica albuginea (mm²)	Area of the corpus cavernosum - without tunica albuginea (mm²)	Area of the tunica albuginea (mm²)	Connective tissue Sv (%)	Sinusoidal space Sv (%)	Smooth muscle fibers Sv (%)	Elastic system fibers Sv (%)
C1	289.5	327.0	5.08	3.46	2.35	1.11	47.76	29.00	22.24	10.00
C2	291.0	322.0	4.38	3.06	1.94	1.12	45.56	30.16	23.28	14.45
C3	277.0	312.5	4.42	3.04	1.91	1.13	46.96	31.76	20.28	16.39
C4	281.5	315.0	4.69	3.25	1.41	1.34	51.40	22.64	22.57	15.14
C5	294.0	323.5	4.59	3.18	1.86	1.34	42.20	34.56	22.04	12.87
C6	272.5	318.0	6.22	4.14	1.34	1.92	42.72	34.24	22.04	8.60
C7	281.0	321.0	5.17	3.55	1.34	1.47	50.88	29.12	19.00	11.15
C8	302.5	329.5	4.52	3.18	1.92	1.31	51.16	21.36	26.46	14.75
C9	293.0	327.0	4.98	3.41	1.47	1.41	42.68	34.48	21.84	9.65
C10	284.0	319.5	4.38	3.02	1.31	1.16	43.08	34.44	21.48	11.4
Animals from Dutasteride Group	Initial body weight (g)	Final body weight (g)	Cross-sectional penile area (mm²)	Area of the corpus cavernosum - including tunica albuginea (mm²)	Area of the corpus cavernosum - without tunica albuginea (mm²)	Area of the tunica albuginea (mm²)	Connective tissue Sv (%)	Sinusoidal space Sv (%)	Smooth muscle fibers Sv (%)	Elastic system fibers Sv (%)
D1	289.5	325.0	4.33	2.92	1.94	0.973	65.92	21.68	11.04	19.50
D2	286.5	337.0	4.01	2.69	1.70	0.984	60.80	25.48	12.72	21.25
D3	233.5	351.0	4.24	2.89	1.96	0.933	68.68	22.20	8.12	19.60
D4	299.5	322.0	4.00	2.73	1.83	0.901	66.44	20.20	12.36	23.56
D5	284.5	337.5	3.92	2.78	1.91	1.020	69.24	19.76	10.00	19.75
D6	283.0	333.0	3.60	2.30	1.89	0.995	72.36	16.36	10.28	17.00
D7	315.0	334.0	4.31	3.00	2.04	0.961	68.88	19.48	10.64	16.60
D8	296.0	339.5	3.70	2.82	1.96	0.866	71.04	17.56	10.40	17.64
D9	303.5	334.5	4.22	2.80	1.84	0.961	64.08	23.60	11.32	18.15
D10	286.5	320.0	4.17	2.94	1.95	0.990	59.44	27.44	12.12	19.47
Animals from Tamsulosin Group	Initial body weight (g)	Final body weight (g)	Cross-sectional penile area (mm²)	Area of the corpus cavernosum - including tunica albuginea (mm²)	Area of the corpus cavernosum - without tunica albuginea (mm²)	Area of the tunica albuginea (mm²)	Connective tissue Sv (%)	Sinusoidal space Sv (%)	Smooth muscle fibers Sv (%)	Elastic system fibers Sv (%)
T1	295.0	327.0	3.94	2.57	1.46	1.10	60.24	22.92	15.84	14.3
T2	290.5	331.5	4.00	2.76	1.75	1.01	62.56	20.36	16.08	12.00
T3	281.0	328.0	4.11	3.22	2.09	1.12	53.84	27.20	17.96	16.15
T4	310.0	349.0	4.09	3.16	2.12	1.05	73.32	15.80	9.88	11.81
T5	282.5	323.0	4.77	3.22	1.89	1.33	57.52	26.48	15.00	13.10
T6	285.4	306.0	3.65	2.48	1.57	0.915	59.84	22.96	16.20	14.50
T7	276.5	321.0	4.24	3.06	1.87	1.19	66.04	18.84	14.12	13.55
T8	285.0	317.0	3.63	2.55	1.63	0.921	60.84	19.72	18.44	17.00
T9	292.5	321.0	3.37	2.52	1.54	0.987	58.76	24.92	15.32	15.80
T10	282.0	324.5								

Animals from Dutasteride plus Tamsulosin Group	Initial body weight (g)	Final body weight (g)	Cross-sectional penile area (mm²)	Area of the corpus cavernosum - including tunica albuginea (mm²)	Area of the corpus cavernosum - without tunica albuginea (mm²)	Area of the tunica albuginea (mm²)	Connective tissue Sv (%)	Sinusoidal space Sv (%)	Smooth muscle fibers Sv (%)	Elastic system fibers Sv (%)
DT1	294.5	315.0	3.69	2.63	1.78	0.85	66.68	23.04	9.28	21.15
DT2	309.5	329.0	3.90	2.54	1.56	0.986	65.24	22.76	11.00	22.00
DT3	319.0	356.0	3.59	2.11	1.33	0.803	73.68	16.76	8.56	19.70
DT4	289.5	309.0	3.76	2.46	1.41	1.05	71.84	17.36	9.80	14.25
DT5	288.0	337.0	3.37	2.37	1.64	0.733	66.20	20.04	12.76	19.80
DT6	287.0	305.5	3.30	2.16	1.67	0.892	75.00	14.52	9.48	21.40
DT7	284.5	297.5	3.45	2.41	1.57	0.843	70.28	19.92	8.80	21.50
DT8	270.5	328.5	3.26	2.47	1.53	0.937	71.72	17.76	9.52	18.63
DT9	298.0	322.5								
DT10	293.0	321.0								
